# Recent advances in microbial and enzymatic engineering for the biodegradation of micro- and nanoplastics

**DOI:** 10.1039/d4ra00844h

**Published:** 2024-03-25

**Authors:** Jaewon Choi, Hongbin Kim, Yu-Rim Ahn, Minse Kim, Seona Yu, Nanhyeon Kim, Su Yeon Lim, Jeong-Ann Park, Suk-Jin Ha, Kwang Suk Lim, Hyun-Ouk Kim

**Affiliations:** a Division of Chemical Engineering and Bioengineering, College of Art, Culture and Engineering, Kangwon National University Chuncheon Korea kimhoman@kangwon.ac.kr kslim@kangwon.ac.kr; b Department of Smart Health Science and Technology, Kangwon National University Chuncheon Korea; c Department of Environmental Engineering, Kangwon National University Chuncheon 24341 Republic of Korea

## Abstract

This review examines the escalating issue of plastic pollution, specifically highlighting the detrimental effects on the environment and human health caused by microplastics and nanoplastics. The extensive use of synthetic polymers such as polyethylene (PE), polyethylene terephthalate (PET), and polystyrene (PS) has raised significant environmental concerns because of their long-lasting and non-degradable characteristics. This review delves into the role of enzymatic and microbial strategies in breaking down these polymers, showcasing recent advancements in the field. The intricacies of enzymatic degradation are thoroughly examined, including the effectiveness of enzymes such as PETase and MHETase, as well as the contribution of microbial pathways in breaking down resilient polymers into more benign substances. The paper also discusses the impact of chemical composition on plastic degradation kinetics and emphasizes the need for an approach to managing the environmental impact of synthetic polymers. The review highlights the significance of comprehending the physical characteristics and long-term impacts of micro- and nanoplastics in different ecosystems. Furthermore, it points out the environmental and health consequences of these contaminants, such as their ability to cause cancer and interfere with the endocrine system. The paper emphasizes the need for advanced analytical methods and effective strategies for enzymatic degradation, as well as continued research and development in this area. This review highlights the crucial role of enzymatic and microbial strategies in addressing plastic pollution and proposes methods to create effective and environmentally friendly solutions.

## Abstract 2

This review examines the escalating issue of plastic pollution, specifically highlighting the detrimental effects on the environment and human health caused by microplastics and nanoplastics.

## Graphical abstract

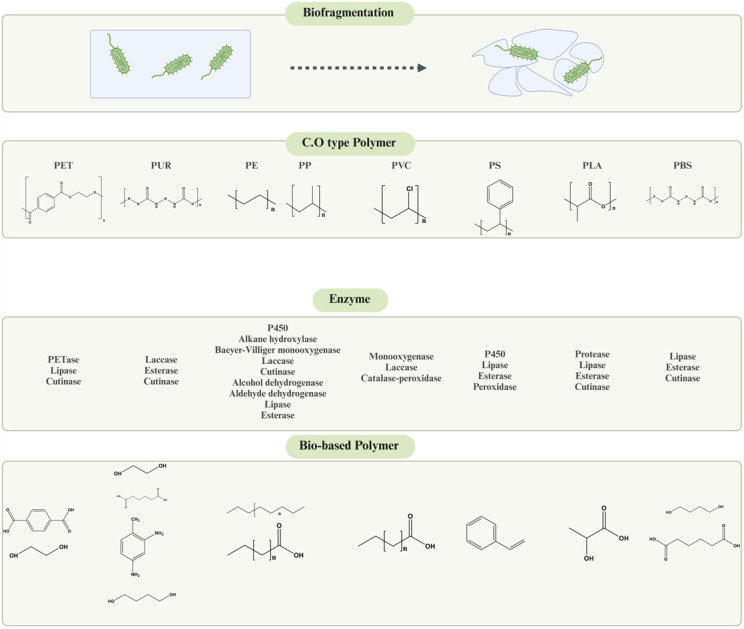

## Introduction

Plastic, first introduced in the mid-20th century, has been essential to contemporary civilization due to its convenience and cost-effectiveness. Nevertheless, its extensive use has resulted in notable difficulties in waste management.^1^ Projections indicate that the worldwide output of plastic might exceed 8.3 billion tons, possibly leading to a concerning 12 billion tons of garbage by the year 2050.^2^ The buildup of waste in landfills and natural ecosystems poses significant environmental issues. The prevailing pattern highlights the need for sustainable solutions, given that conventional approaches such as recycling and burning have shown their insufficiency, resulting in a substantial buildup of plastic trash in landfills.^3^ The magnitude of plastic manufacturing, coupled with inadequate waste disposal methods, has resulted in the pervasive presence of plastic trash and subsequent environmental pollution caused by microplastics, which are formed as a consequence of the degradation of larger plastic objects.^3,4^ These present substantial hazards to ecosystems, human well-being, and safety.^5,6^ The increasing recognition of this ecological problem emphasizes the pressing need for effective degradation remedies.

The widespread manufacturing and disposal of plastics have resulted in substantial contamination in land, water, and air environments, with microplastics being especially abundant. Inadequate waste management on land is a significant cause of marine plastic pollution, leading to an estimated 5.25 trillion microplastics and nanoplastics entering seas, soil, and air.^7^ The presence of these minuscule particles is worrisome because of their detrimental impact on soil fertility and the well-being of marine organisms. Microplastics and nanoplastics have a profound negative impact on ecosystems and the health of species. They disrupt natural processes, damage animals, and accumulate toxic compounds in their organs.^8^ The presence of microplastics has the potential to affect the capacity of soil to allow water to pass through, its density, and the movement of nutrients, which raises worries about the consequences of their presence.^9,10^ The entrance of micro- and nanoplastics into oceans is influenced by several processes, including UV photodegradation, mechanical forces, hydrolysis, and biological degradation.^11,12^ These minuscule particles disperse extensively across the ocean environment and have a notable influence on marine life and ecosystems. Due to their diminutive size, they can effortlessly infiltrate organisms and accumulate inside their organs, resulting in the accumulation of detrimental compounds that cause significant hazards to marine life.^13^

**Scheme 1:**
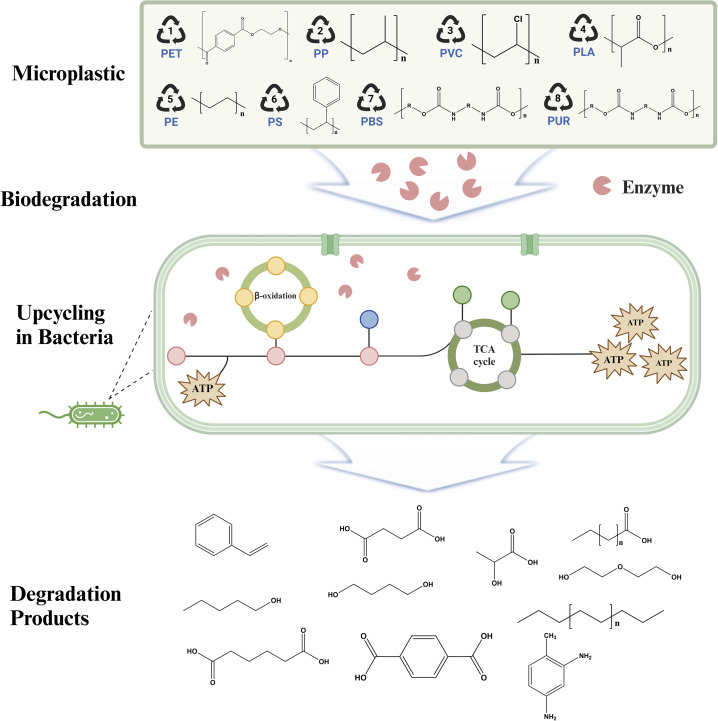
Schematic diagram of microbial and enzymatic degradation and upcycling of microplastic. Microplastics in the environment undergo enzymatic degradation by extracellular enzymes and are then utilized as a carbon source by microorganisms, ultimately leading to complete. Figures generated with BioRender (https://biorender.com/).

The widespread use of artificial polymers, which are crucial in contemporary society, has reached concerning levels and poses substantial ecological and physiological hazards in terrestrial, aquatic, and marine ecosystems. Due to their small size and large surface area, microplastics are very susceptible to absorbing harmful compounds including heavy metals, medicines, and flame retardants.^14^ Additionally, they are more easily consumed by living creatures. These activities result in a range of negative consequences for plants and animals, such as alterations in soil composition, increased growth of microorganisms, inflammation in fish, compromised immune systems, reproductive problems, and malfunction in organs.^15–19^ The heightened vulnerability of humans makes them particularly susceptible to the substantial risk posed by microplastic pollution. The pollutants have the ability to initiate inflammatory and neurotoxic effects by activating certain protein kinase pathways. The effects of microplastics among various creatures differ depending on their ability to withstand environmental stress and the ecological circumstances in which they live.^20,21^ The extensive use of these polymers and their inclination to amass contaminants emphasize the need for devising efficient degrading techniques. The aim of these techniques is to disassemble microplastics and reduce their presence in the environment and alleviate the harm they cause (Scheme 1).

Plastics including polyethylene (PE), polyethylene terephthalate (PET), polyurethane (PU), polystyrene (PS), polypropylene (PP), and polyvinyl chloride (PVC) have notable environmental difficulties since they degrade slowly in nature.^22^ The degradation pathways of these polymers may be classified according to their chemical composition: those with a carbon–carbon backbone and those with heteroatoms in the main chain.^23,24^ The focus of this review is on the methodologies used by microorganisms to degrade synthetic polymers and the specific roles of various enzymes in this biological process. These technologies provide effective methods for transforming plastic trash into carbon atoms, carbon dioxide, and valuable chemicals. The use of enzyme technology is crucial in promoting environmental conservation by converting plastic waste into less detrimental compounds.^25^ This review will explore the most recent developments in enzymatic degradation techniques and assess their potential in reducing the environmental and health consequences of plastic pollution, with a specific emphasis on microplastics and nanoplastics.

## The longevity and obstacles of synthetic polymers in the environment

### Enzymatic degradation challenges and progress in common synthetic polymers

The extensive use of synthetic polymers such as PET, PE, PS, PP, and PVC has played a substantial role in the global surge of plastic production, which reached a staggering 335 million tons in 2016.^26^ These materials, which are used in numerous industries, present significant environmental obstacles because of their long-lasting and non-degradable characteristics.^4^ The increasing use of these polymers in our everyday lives calls for a careful evaluation of their environmental consequences, specifically in terms of energy usage and waste production.^27^ This situation has prompted a greater focus on the development of sustainable alternatives and recycling methods. The pressing concern lies in finding innovative solutions that can mitigate the adverse effects of plastic proliferation, while maintaining a balance between the utility of these polymers and their environmental footprint.

The conversation surrounding the use of synthetic polymers revolves around their essential role in contemporary society and the resulting environmental hurdles. There are ongoing efforts to investigate environmentally friendly materials and improve recycling methods, but these endeavors encounter various obstacles, such as technological constraints and economic viability.^28^ The management of synthetic polymers' impact on the environment is a multifaceted challenge that necessitates a comprehensive approach.

### Polyethylene (PE)

Dealing with polyethylene poses a serious challenge for enzymes when it comes to breaking down microplastics. This material is often used in packaging and containers because of its outstanding ability to resist microbial degradation. PE, especially HDPE, possesses a linear structure that enhances its resistance to enzymatic degradation. Exciting advancements in biotechnology have revealed the incredible capabilities of specific enzymes and microbial strains in effectively binding to and breaking down PE.^29–31^ Usually, these enzymes work by oxidizing the polymer chains, initiating a process of breaking them apart.^32^ Several environmental factors, such as exposure to UV light or physical abrasion, can contribute to this process.^33^ These factors enhance the surface area to promote enzymatic activity.

### Polyethylene terephthalate (PETE/PET)

**Fig. 1:**
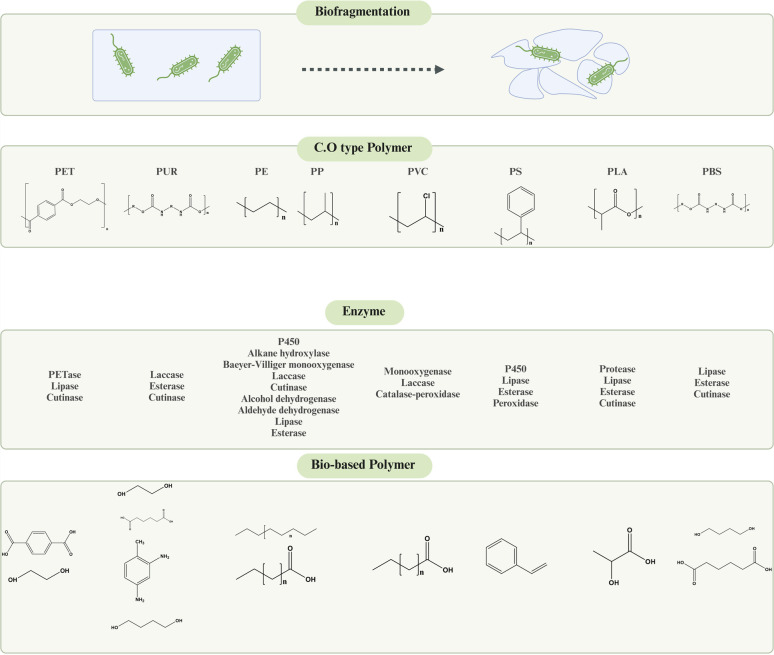
Enzymatic degradation of various types of plastics. Microorganisms inhabiting various surfaces of microplastics release various types of extracellular enzymes for biodegradation and fragmentation. Figures generated with BioRender (https://biorender.com/).

Extensive research has been conducted on the enzymatic degradation of PET, which is commonly found in beverage bottles and textiles. Researchers have made notable discoveries in the area of enzyme research and revealed the impressive capabilities of enzymes such as PETase and MHETase in efficiently breaking down PET into its fundamental components, ethylene glycol and terephthalic acid.^34–36^ These enzymes have been found in certain bacteria and fungi and their efficiency and longevity are being enhanced for industrial use.^37–39^ The potential of enzymatic degradation of PET for recycling operations is immense and offers a more environmentally friendly and efficient option compared to traditional mechanical and chemical recycling methods (Fig. 1).

### Polypropylene (PP)

PP is widely used in packaging and automotive components due to its impressive resistance to a range of chemical degradation processes.^40,41^ However, a recent study has uncovered the potential of enzymatic techniques in degrading PP. Certain microbial strains have been acknowledged for their remarkable capacity to adhere to and partially degrade PP.^42,43^ The challenge is to enhance the efficiency of these biological processes to make them more practical for widespread use. A study is being conducted to explore and improve enzymes that have the potential to enhance the efficiency of breaking down the chemical bonds in PP.^44^

### Polyvinyl chloride (PVC)

PVC, widely used in the construction and packaging industries, poses challenges for biodegradation because of its chlorine content.^45^ There is a lack of comprehensive scientific knowledge regarding the enzymatic degradation of PVC, especially when compared to other polymers. There has been a strong focus on identifying and managing microbial strains that can tolerate and break down the chlorine-based compounds commonly found in PVC.^46,47^ The study in this area is still in its initial phases with the goal of developing dependable and effective methods for the secure breakdown of PVC.

### Polystyrene (PS)

PS, a widely used material in packaging and insulation, is well-known for its remarkable durability due to its chemically stable aromatic structure.^48,49^ However, recent discoveries have revealed the presence of certain bacteria and fungi that can degrade PS using specialized enzymes.^50–52^ The enzymes effectively break down the styrene monomers, and the current pace and efficiency of the process meets expectations.^53^ The present study aims to enhance the efficiency and longevity of these enzymes to make the biodegradation of PS a more practical option.

### Polylactic acid (PLA)

PLA, a bioplastic derived from renewable sources, is inherently more prone to enzymatic degradation when compared to polymers made from petroleum.^54^ Research has shown that certain enzymes, such as proteinase K and lipase, have proven to be extremely efficient in the degradation of PLA.^55–57^ The degradation of PLA happens through the hydrolysis of ester bonds, leading to the formation of lactic acid, which can be subsequently metabolized by various microorganisms.^58^ PLA stands out for its positive environmental impact since it can be easily and completely broken down in composting facilities.

### Polybutylene succinate (PBS)

It has been observed that PBS, a type of biodegradable plastic, is more susceptible to enzymatic degradation compared to conventional polymers.^2,59^ There have been significant findings in the field of enzyme research, particularly in the area of PBS degradation.^60,61^ Notably, lipases have demonstrated remarkable efficiency in breaking down PBS. The degradation process involves the hydrolysis of ester bonds in the polymer, causing it to break down into smaller particles that can be easily broken down by biological processes.^62^ Because of its remarkable vulnerability to degradation, PBS is an extremely appealing material for endeavors that prioritize ecological sustainability.

### Polyurethane (PUR)

Enzymatic degradation of PUR can be quite complex due to its various chemical compositions.^63,64^ However, certain enzymes such as esterase and ureases show great potential in breaking down PUR.^28,65,66^ The enzymes have the ability to specifically target certain bonds in the PUR polymer, resulting in its degradation.^28^ Researchers in this field are focused on uncovering and enhancing the efficiency of these enzymes with the goal of developing effective techniques for breaking down PURs. The significance of this issue stems from the widespread use and persistent presence of PURs in the environment.

### Influence of chemical composition on plastic degradation dynamics

The dynamics of plastic degradation are closely connected to various internal and external factors, particularly in relation to enzymatic activity on microplastics.^67–69^ These factors encompass the intrinsic characteristics of the plastic, such as its molecular structure, composition, physical form, and the inclusion of additives.^23,70,71^ The rate and mechanism of biodegradation are greatly influenced by these characteristics. Note that the susceptibility of a plastic to enzymatic attack can be significantly affected by the presence of specific functional groups or the degree of polymer branching.

Factors such as pH, temperature, oxygen levels, and light exposure are critical in influencing the effectiveness of enzymatic degradation. Certain enzymes exhibit improved performance or durability in particular environmental conditions, which can impact the rate of degradation.^72–75^ Environmental factors can also weaken the structure of plastics, making them more susceptible to enzymatic breakdown.

The fundamental chemical composition of plastics, specifically the types of bonds they possess, plays a crucial role in determining their degradability. Plastics with carbon–carbon (C–C) backbones, such as polyethylene and polypropylene, have a remarkable ability to resist microbial and enzymatic decomposition, which significantly slows down the degradation process.^76^ On the other hand, polymers that incorporate heteroatoms into their main chain, such as polyesters, are highly prone to enzymatic hydrolysis.^77^

The interaction between degrading enzymes and a plastic is greatly influenced by the chemical composition, which determines the surface hydrophobicity. Surfaces that repel water can also cause hydrophilic enzymes to be repelled, which presents initial obstacles in the biodegradation process. Effective enzymatic binding and action often require the formation of a biofilm.^78,79^

Given the origin of most modern plastics from petrochemical sources, the widespread existence of non-biodegradable plastics presents a notable environmental concern.^13^ To tackle this problem, it is important to keep working on improving our understanding and finding ways to enhance the degradation of microplastics by enzymes. Adapting enzymatic methods to effectively tackle the distinct obstacles posed by various plastic polymers is needed for advancing sustainable waste management and environmental conservation strategies.

### Examining the enduring environmental effects of microplastics and nanoplastics

It is essential to have a comprehensive understanding of the physical characteristics and long-term effects of microplastics (MPs) and nanoplastics (NPs) in order to effectively tackle the ongoing issue of these pollutants in various ecosystems. The extensive variety of synthetic polymers used in the composition of MPs results in a wide range of physical forms, including foam, pellets, flakes, fibers, and films. It is important to note that a significant portion of these particles, approximately 60%, are smaller than 1 mm in size, primarily appearing as flakes and fibers.^80–84^ Nanoplastics possess distinct characteristics in comparison to MPs as a result of their reduced dimensions. The difference in size has a notable impact on their movement, behavior in the environment, interactions, availability to organisms, and potential harm.^85–88^

Both industrial and residential activities contribute to the release of these pollutants, leading to the contamination of aquatic systems, air, and soil.^89–91^ The widespread pollution poses considerable threats to multiple aspects of the ecosystem, such as the food chain, plant life, marine organisms, and human populations.^92,93^ This pollution greatly affects both humans and the environment, as they both experience the consequences of it. The structural composition of polymers plays a key role in determining their environmental behavior and has a combination of ordered and disordered regions that have a significant impact.^23^ The current situation underscores the need for effective methods to address these persistent pollutants.

### Exploring the ecological and health implications of microplastic and nanoplastic pollution

The presence of MPs and NPs in different ecosystems, whether due to natural processes such as erosion or human activities such as industrial emissions, results in a wide range of harmful consequences.^94–96^ These particles have been associated with serious health risks, such as the potential to cause cancer and interfere with endocrine systems.^97,98^ Their environmental impact is exacerbated by their capacity to attract and transport other detrimental substances, including persistent organic pollutants and heavy metals.^99,100^ In addition, MPs and NPs can carry harmful bacteria, including antibiotic-resistant strains, which poses a considerable threat to both wildlife and human communities.

The management and regulation of MPs and NPs require advanced analytical and quantitative methods, particularly when addressing complex environmental matrices. The challenge is made more difficult by the complexity of recycling these particles.^101^ A deep understanding of the physical and chemical properties of MPs and NPs is needed to develop effective strategies for their enzymatic degradation.

In order to effectively tackle the negative impacts of MPs and NPs, it is important to acknowledge their wide-ranging implications and establish dependable approaches for their identification, assessment, and breakdown. This field of study not only provides potential solutions for addressing the environmental and health risks linked to microplastic and nanoplastic pollution, but also emphasizes the importance of ongoing efforts to comprehend and combat this widespread problem.

## The role of microbes in plastic degradation

### The crucial role of microorganisms in plastic degradation

As we strive to find sustainable and environmentally friendly solutions to the problem of plastic pollution, the importance of microorganisms in breaking down microplastics (MPs) and nanoplastics (NPs) has become a significant focus.^102–106^ This area of study investigates the capacity of different microorganisms and insects to transform plastic into eco-friendly substances.^92,93^ Several microorganisms, such as bacteria, fungi, and actinomycetes, have been recognized for their enzymatic capability to degrade the intricate chemical bonds of microplastics into simpler compounds.^107–109^

These microorganisms, found in various habitats such as soil, aquatic environments, and even air, have an important role in the natural breakdown of organic matter. An excellent illustration is the breakdown of PE, a commonly used plastic, by a range of microorganisms.^29,31^ One of the most fascinating findings in this field involves the gut bacteria found in the larvae of the large waxworm, *Galleria mellonella*.^110,111^ These bacteria have demonstrated impressive effectiveness in breaking down PE through a process known as hydrolysis.^112^

This research on the interaction between microbes and plastic has uncovered exciting possibilities for addressing plastic waste. It has the potential to transform discarded petroleum-based polymers into reusable materials or feedstock for biomass production, and offers new ways to manage plastic waste effectively.^24,113^ The success of these groundbreaking solutions depends on a comprehensive grasp of the distinct microbial enzymes implicated in the degradation process. A deep understanding of this subject is crucial to create more efficient and sustainable approaches to tackle the growing issue of plastic pollution and advance the field of enzyme-based microplastic degradation.

### Dynamics of the microbial degradation of plastics

**Fig. 2:**
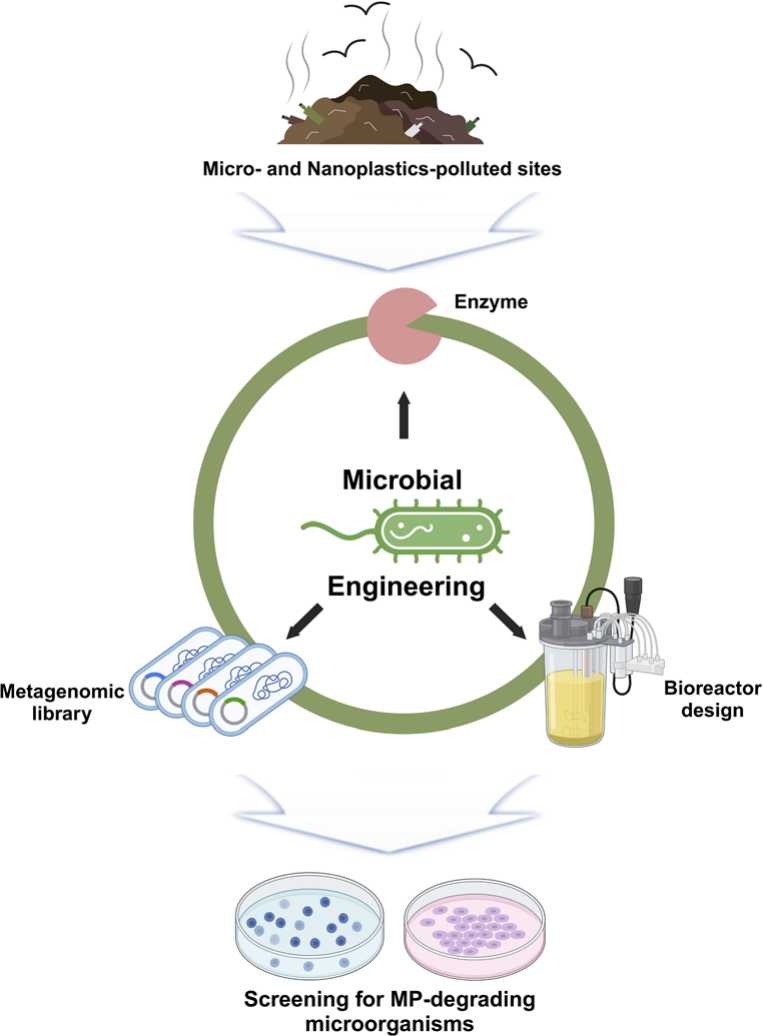
Schematic diagram of the increase in microplastic decomposition efficiency using microbial engineering. Microorganisms discovered in various waste treatment facilities are ultimately improved for efficient microplastic degradation through various processes such as protein engineering, metagenomic library techniques, and bioreactor design. Figures generated with BioRender (https://biorender.com/).

The breakdown of microplastics (MPs) by microorganisms is a sophisticated process driven by enzymes. Many factors can influence the process, including the molecular weight and chemical composition of the microplastic, environmental conditions, the specific microbial species, and the physical properties of the plastic, such as crystallinity and the presence of functional groups or additives.^8,9^ The degradation process usually occurs in a sequence of stages beginning with the development of a biofilm on the surface of the plastic. This is then followed by biodegradation, biotransformation, and finally mineralization.^35,114^ Irrespective of the natural degradability of the polymer, the first step involves hydrolysis, which breaks down microplastics into smaller molecular fragments. This step is crucial and relies on the microorganisms at hand (Fig. 2).

Microorganisms play a key role in the intricate biochemical reactions that occur during the aerobic biodegradation of plastics. Microorganisms use oxidizing enzymes to generate carbonyl groups, which are subsequently oxidized into carboxylic acids.^115^ This results in the hydrolysis of the polymer chain, which facilitates degradation. The microorganisms metabolize the small hydrocarbon fragments produced in a highly efficient manner.^116^ The last step involves converting the hydrolysis products into microbial biomass, resulting in the release of water and carbon dioxide.^93,117^ The enzymes involved in this degradation process can be classified into two main categories: those that alter the surface of microplastics to enhance their solubility in water, and those that break down the plastic into smaller components for microbial metabolism.^118^

An understanding of the intricate processes involved in the breakdown of microplastics by microorganisms is needed to devise successful approaches to minimize the negative effects of microplastic pollution on the environment. It is also crucial for making progress in enzyme-based techniques for microplastic degradation and gaining valuable insights into potential solutions for this urgent environmental issue (Table 1).

**Table 1:** Types of plastic-degrading microorganisms and their degradation efficiency

Microorganism	Enzyme	Temperature range (°C)	Biological effects (results)	Ref.
**Polyethylene terephthalate**				
*Ideonella sakaiensis* 201-F6	PETase	20∼45	Almost completely degraded after 6 weeks	34
*Thermobifida fusca* DSM43793	TfH	30–60	50% degraded after 3 weeks	119
*Fusarium solani* pisi	FsC	30–60	97% weight loss after 96 hours	120
*Thermobifida cellulosilytica* DSM44535	Thc_Cut1	50	Increase of reactive hydroxyl or carboxyl groups	121
*Saccharomonospora viridis* AHK190	Cut190 S226P/R228S	60–65	27% weight loss after 3 days	122
*Bacillus subtilis* 4P3-11	BsEstB	40–45	Introduction of novel carboxyl and hydroxyl groups	123
*Thermomonospora curvata* DSM 43183	Tcur0390	50	Stronger substrate affinity and increase of the H–S distance	124

**Polypropylene**				
*Pseudomonas aeruginosa* WGH-6	AH alkane hydroxylase	30	17.2% weight loss after 40 days	44
*Aneurinibacillus* spp.	Lipase	50	44.2% weight loss after 140 days	125
*Brevibacillus* spp.				

**Polyethylene**				
*Microbacterium paraoxydans*	Lac	Room temperature	61% weight loss after 2 months	126
*Alternaria alternata* FB1	153 potential enzymes	30	62.79% decreased after 28 days	127

**Polyvinyl chloride**				
*Klebsiella* sp. EMBL-1	Catalase-peroxidase	30	19.57% weight loss after 3 months	46

**Polyurethane**				
*Rhodococcus equi* TB-60	Urethane hydrolase	30	70% degradation after 10 days	128

**Polystyrene**				
*Pseudomonas aeruginosa* DSM 50071	SGT/SH	25	WCA decreased from 91.56° to 79.8° after 2 months	129
*Bacillus paralicheniformis* G1	Alkane monooxygenase/cytochrome P450	30	34% weight loss after 2 months	53

### Determinants of microbial efficacy in plastic degradation

The degradation process of microplastics is influenced by a variety of factors including microbial growth kinetics, the properties of microplastics themselves, and the environmental conditions at hand. The structural properties of microplastics, their material composition, shape, and the presence of additives play a main role in determining their vulnerability to biodegradation.

External environmental factors, including pH levels, temperature, oxygen availability, exposure to light, and the presence of other substances, are crucial in influencing the process. The interaction between temperature and pH is key since pH levels have a significant impact on the electrostatic interactions between the microplastic surface and microorganisms and chemicals in soil or water.^130^ Degradation can be slowed down under certain conditions, while enzyme activity may be affected in different environments. For the most effective biological degradation of microplastics, it is important to maintain optimal conditions such as low pH levels and low temperatures. The rate of degradation can be influenced by a range of chemical and physical properties of microplastics, including density, molecular weight, degree of crystallinity, and the presence of specific functional groups or substituents.^131^ Microplastics with carbon–carbon (C–C) bonds provide enhanced durability against microbial attack, whereas microplastics with ester bonds are more prone to the effects of hydrolytic enzymes.^116^

**Fig. 3:**
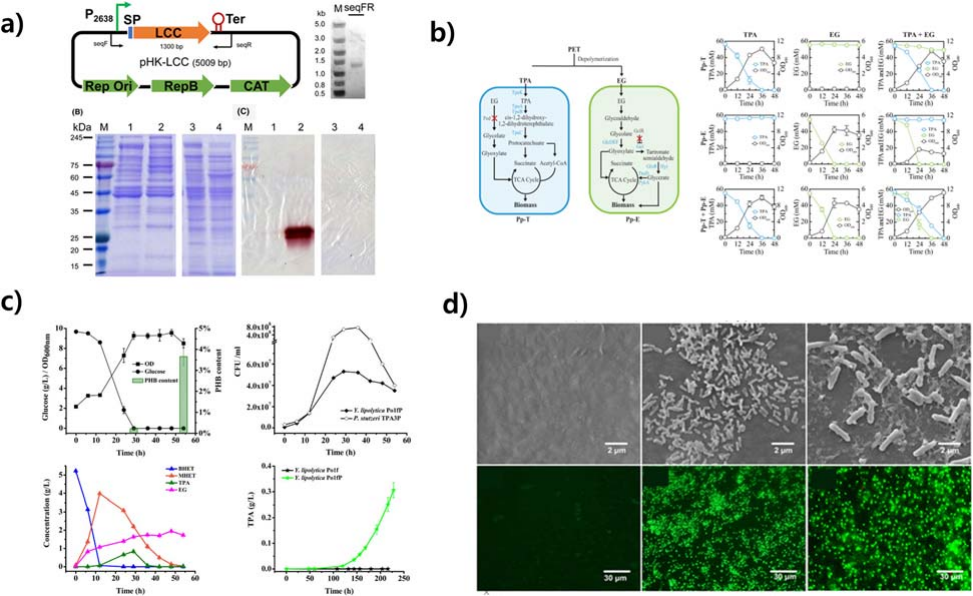
Microplastic decomposition using microbial engineering. (a) Construction of LCC-expressing plasmid pHK-LCC and extracellular expression of active LCC in *C. thermocellum*. Reproduced with permission from ref. 36. Copyright (2020) John Wiley & Sons, Inc. (b) Schematic of the designer T–E consortium composed of two strains Pp-T and Pp-E. Pp-T specializes in TPA degradation, which was developed by deleting the ped operon and constitutively expressing the genes tpaAa, tpaAb, tpaB, tpaC, and tpaK. Reproduced with permission from ref. 132. Copyright (2023) Springer Nature. (c) Co-cultivation of *Y. lipolytica* Po1fP and *P. stutzeri* TPA3P. (A) OD, glucose consumption, and PHB content. (B) BHET hydrolysis curve. Reproduced with permission from ref. 133. Copyright (2021) Elsevier. (d) Biofilm formation and viability. Morphotypes of the cells in the mature biofilm on the PE sheet. Fluorescent microscopic images of biofilms, which show cell viability after the 28 day incubation. Reproduced with permission from ref. 110. Copyright (2014) American Chemical Society.

The inclusion of plasticizers or other additives can greatly affect the biodegradability of microplastics. These additives have the potential to either enhance or impede microbial colonization depending on their unique characteristics and the composition of the microbial community they are targeting.^116^ The presence of external substances on the surface of microplastics can slow microbial degradation. On the other hand, nutritional supplements that are high in carbon and nitrogen can enhance the growth of microorganisms on microplastic surfaces, thereby speeding up the degradation process.^131^ These different factors are important in the development of enzyme-based approaches for efficient microplastic degradation (Fig. 3).

### Evaluating the effectiveness and challenges of biological plastic degradation

Progress in the degradation of plastics by microorganisms, particularly with enzyme-based methods, has been impressive. Biocatalysts have demonstrated significant potential in breaking down microplastics (MP) into smaller particles such as nanoplastics (NP). There is increasing interest in these biocatalysts due to their ability to greatly decrease the size of plastic particles. Nevertheless, the efficiency of microorganisms in degrading plastics is influenced by the various characteristics of the materials and is contingent upon the size of the plastic particles, ranging from larger flakes to minuscule dimensions.^134^

Several biodegradation methods are currently under investigation, such as pure bacterial cultures, fungal cultures, bacterial consortia, and the use of specific enzymes.^135^ Microorganisms such as bacteria, fungi, and algae have shown the ability to break down complex plastic polymer chains into smaller units, or monomers.^136,137^ This ability is derived from the production of certain enzymes or metabolites that aid in the degradation process. One of the difficulties of microbial degradation is the considerable amount of time needed to break down contaminants. Even with the demonstrated efficacy of bacteria, the process of breaking down microplastics can be time-consuming in certain instances and often takes several months. Efforts are currently being made to enhance environmental conditions and optimize microbial strains to accelerate the degradation process and improve the efficiency of bacterial action on MPs and NPs. In light of the difficulties posed by degradation rates, there are continuous endeavors to investigate the thorough biodegradation of microplastics and nanoplastics.

## Approaches for plastic degradation using enzymes

### The crucial role of microorganisms in plastic degradation

The issue of plastic pollution has become more pressing, and the development of creative and environmentally friendly solutions, particularly in the area of plastic degradation, is needed. Enzymatic methods for plastic degradation have become increasingly recognized as very effective and eco-friendly alternatives to traditional plastic treatment methods. This strategy, grounded in a commitment to environmental sustainability, offers a way to transform plastics into reusable monomers or create valuable bioproducts by converting them into carbon dioxide, water, and new biomass.^138,139^

Microorganisms are crucial in this process since they produce specialized enzymes that can effectively break down polymers and support the metabolism of the resulting hydrolyzates. The enzymatic biodegradation of polymers usually involves important reactions such as hydrolysis and oxidation, which break down polymer chains into smaller oligomers and monomers.^140,141^ These reactions are essential for the breakdown of various polymer bonds, such as ester, carbonate, amide, and glycoside bonds, resulting in the creation of monomers. In terms of their susceptibility to enzymatic degradation, petroleum-based polymers can be categorized into two types: hydrolyzable and non-hydrolyzable. Examples of hydrolyzable polymers include polyethylene terephthalate (PET) and polyurethane (PUR), while non-hydrolyzable polymers include polyethylene (PE), polystyrene (PS), polypropylene (PP), and polyvinyl chloride (PVC).^141,142^ Enzymes from the hydrolytic enzyme group, including esterases, lipases, depolymerizers, and fetases, have a high proficiency in breaking down the carbon structure of plastics. They focus on the ester bonds and carbonyl carbon atoms that are created when the oxidation process occurs, transforming the polymer into individual monomers.^143^

Nevertheless, the degradation of non-hydrolyzable polymers such as PE, PS, PP, and PVC poses a considerable obstacle. The chemical degradation of the carbon backbone is a complex area that still requires further research. Ongoing progress in enzymatic degradation is needed to preserve the environment and improve recycling techniques.

### Enzyme specificity in plastic polymer degradation

**Fig. 4:**
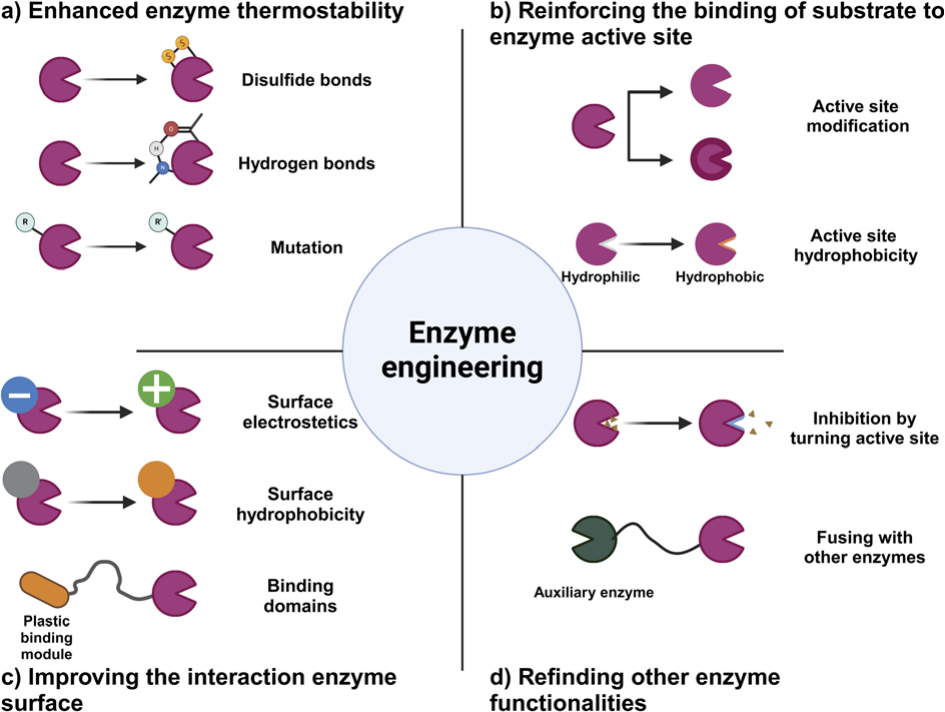
Schematic diagram of protein engineering strategies employed in the modification of enzymes involved in plastic degradation. The incorporation of protein engineering in the enhancement of plastic-degrading enzymes can induce alterations in enzyme activity and characteristics *via* diverse mechanisms. This encompasses the refinement of thermal stability, augmentation of enzyme activity through electrostatic interactions between the enzyme and substrate, and the potentiation of enzyme activity through the integration of accessory.

In the area of enzyme-mediated microplastic degradation, past research has revealed the involvement of numerous microorganisms and enzymes in the breakdown of polyethylene (PE). These organisms, such as actinomycetes, bacteria, and fungi, play distinct roles in the degradation of PE.^144^ Prominent enzymes have been identified as having a crucial role in the process, including manganese peroxidase from fungi such as *Phanerochaete chrysosporium* and *Trametes versicolor*, and alkane hydrolase from *Pseudomonas* species (Fig. 4).^145–147^

The degradation mechanisms for various forms of PE, including LDPE and HDPE, are currently being studied. Enzymes like laccase and alkane hydrolases have demonstrated potential in initiating PE degradation. Laccase, in particular, aids in oxidizing the PE surface and creating carbonyl regions that are more prone to subsequent enzymatic activity. Alkane hydrolases have shown great efficacy in breaking down heat-treated PE.^135,148–151^ Enzymes involved in PE degradation have been isolated from a wide range of microbial sources, including Proteobacteria, Firmicutes, and Actinobacteria. These microorganisms have shown promise in aiding the breakdown of microplastics. However, there is still much to learn about the exact process by which these enzymes degrade microplastics, and further research is needed.^152^ Furthermore, there have been notable advancements in the field of microbial enzymes that possess the ability to break down lignin polymers containing oxidative C–C bonds. Notable examples include manganese peroxidase, lignin peroxidase, and laccase. The understanding of these enzymes in the area of polyethylene biodegradation is continuously developing, and additional research is necessary to gain a complete grasp of their functions and mechanisms. It is important that future studies focus on gaining a deeper understanding and characterization of the different enzymes involved in polyethylene degradation.^153,154^

### Focused enzymatic activity targeting polyethylene terephthalate

The field of enzymatic microplastic degradation has made significant progress, especially in targeting polyethylene terephthalate (PET). This is based on extensive research on *Thermobifida fusca* hydrolase, which has led to the discovery of various enzymes that can break down PET. Out of all the enzymes, the PETase enzyme from *Ideonella sakaiensis* 201-F6 is particularly^142^ noteworthy for its impressive capacity to effectively degrade PET into various intermediates such as BHET, MHET, and TPA. MHETase, an additional enzyme, carries out further processing of MHET to produce terephthalic acid and ethylene glycol.^34,155^

Recent advancements have resulted in the creation of stronger versions of PETase, which have improved stability and efficiency in breaking down materials. In addition, a 25 kDa suberinase from Streptomyces scabies has demonstrated considerable potential in the degradation of PET.^156,157^ Thorough analyses of the structure and mechanisms have uncovered distinct interactions between PETase, cutinase, and PET, which occur through an induced fit mechanism. PETase is capable of breaking down a wide range of polycyclic aromatic microplastics due to its impressive versatility.^158,159^

Efforts to enhance the efficiency of PETase have involved various techniques such as mutagenesis, overexpression, and the use of microalgae for transformation. These advancements are crucial in tackling the environmental issues caused by PET pollution. This research is a significant advancement in developing enzymatic methods to effectively break down PET microplastics and makes a valuable contribution to environmental remediation endeavors.^160,161^

### Enzymatic strategies for polystyrene degradation

Important advancements have been made in the field of enzymatic microplastic degradation, with a particular focus on polystyrene (PS). Progress in this field is evident from the fact that a wide range of microorganisms, such as bacteria and fungi, have the ability to break down PS. Nevertheless, the precise enzymes responsible for initiating this degradation process remain to be fully understood.^135,162^

Prior research has emphasized the significance of extracellular esterases from *Lentinus tigrinus* in the breakdown of PS. It has also mentioned that polymerases from *Bacillus* and *Pseudomonas* species play a role in the breakdown of PS.^163,164^ In addition, there have been reports on enzymes that are involved in breaking down styrene, such as styrene monooxygenase, styrene oxide isomerase, phenylacetaldehydrogenase, and phenylacetyl coenzyme A ligase.^165–169^ These enzymes are essential for the conversion of PS polymers through a series of reactions. It starts with the transformation of monomers into styrene, followed by oxidation to phenylacetate, and ultimately the integration of phenylacetate into the Krebs cycle.^170^

An in-depth knowledge and thorough analysis of these enzymes is essential to enhance the enzymatic breakdown of PS microplastics. This research explores new possibilities for tackling the environmental issues caused by PS waste and offers creative and eco-friendly solutions for managing PS waste. Efforts to identify and optimize these enzymes are needed to develop effective strategies to address the impacts of PS pollution.

### Advancing enzyme engineering for plastic degradation

#### Strategies for enhancing stability and efficacy of enzymes in plastic degradation

Advances in enzyme engineering are necessary to effectively break down microplastics. An important aspect in this field is to enhance the stability and activity of enzymes, especially those involved in breaking down plastic materials.^171^ This entails leveraging structural similarities between various enzymes to enhance their functional properties, typically with the application of site-directed mutagenesis techniques.^172^

Another important development involves enhancing the heat tolerance of enzymes that target plastics. This is particularly important for plastics with high glass transition temperatures (*T*_g_), as their crystallinity decreases as the temperature rises.^173^ This can make it easier for enzymes to access the plastics and speed up degradation. Plastics are easier to process by enzymes when they become more flexible and mobile at temperatures near or above their *T*_g_.^173^ Nevertheless, a serious challenge arises when it comes to naturally occurring enzymes such as PETase since they tend to lose their efficiency when exposed to high temperatures, which restricts their ability to maintain thermal stability.^174^ To tackle this problem, previous studies have primarily concentrated on developing different versions of PETase and similar enzymes that possess the ability to endure elevated temperatures, thus aligning with the *T*_g_ of various types of plastics. This requires a thorough examination of the distinctive characteristics of thermophilic proteins and using this knowledge to enhance the ability of plastic-degrading enzymes to withstand high temperatures. The ultimate objective is to maximize the efficiency of these enzymes, particularly in environments that closely align with the *T*_g_ of the specific plastic being targeted.^13,122^

With the development of enhanced enzymes, we can create enzymes with greater power and efficiency that enables them to break down a wider variety of plastics. Enhancement of the thermal stability and pH tolerance of these enzymes has the potential to significantly improve their practicality in environmental cleanup and recycling processes. Continual endeavors in enzyme engineering focus on surpassing the constraints of natural enzymes, while also customizing enzymes to fulfil precise needs for plastic degradation.^175^ Our work involves enhancing enzymes to target specific types of plastics, enhancing their selectivity, and maximizing their catalytic efficiency. The improvement of the stability and activity of enzymes is crucial for the progress of enzyme-based microplastic degradation.

### Enhancing enzymatic thermal stability through the use of disulfide bonds for microplastic degradation

The enhancement of enzyme thermal stability has become a key method in enzymatic microplastic degradation, particularly in the area of PET hydrolysis. One effective approach involves incorporating disulfide bonds and salt bridges. This process requires meticulous adjustment of the protein structure at specific locations or in its overall arrangement. As an illustration, the replacement of amino acid residues in metal-binding regions with disulfide bonds has been demonstrated to greatly improve the ability of enzymes to withstand high temperatures.^176^

The significance of disulfide bridges is especially notable in PET hydrolases, which possess numerous sites for binding divalent metals, as evidenced by the crystal structure of the Cut190 enzyme. It has been noted that the inclusion of divalent ions such as calcium (Ca^2+^) or magnesium (Mg^2+^) has the dual effect of enhancing the thermal stability of the enzyme and optimizing its temperature range for operation. Methods such as circular dichroism (CD) have shown that the melting temperature of the enzyme is significantly raised with the addition of calcium ions. Further insights from molecular dynamics (MD) simulations and X-ray structural analysis have uncovered the significance of Ca^2+^ binding in triggering essential conformational alterations in the enzyme, which results in enhanced catalytic efficiency.^122^ The presence of intramolecular disulfide bridges, specifically DS1 and DS2, plays an important role in maintaining the functional integrity of the catalytic triple bond in enzymes such as PETase. One aspect that enhances the flexibility of the loop is DS1, which leads to a boost in enzyme activity. On the other hand, DS2 is crucial in preserving structural stability. Focused enhancement of these sites has been demonstrated to greatly improve the capacity of the enzyme to break down PET.^177,178^ Investigation of the crystal structures of different enzymes, such as LCC, Tf cutinase, and IsPETase, has opened up possibilities for developing innovative approaches to enhance their resistance to heat. As an illustration, enzyme activity has been enhanced by replacing the divalent metal binding site of LCC with a disulfide bridge and introducing targeted mutations.^179^ Currently, there are ongoing efforts to enhance the thermal stability of enzymes that play a role in breaking down plastics, specifically PET. This task requires the generation of enzyme mutants using directed evolution techniques, as well as the identification of optimal sites for calcium binding and disulfide bridge formation.

The strategic use of disulfide bonds is demonstrating the enhancement of the thermal stability of enzymes employed in the degradation of microplastics. These advancements are crucial for the development of stronger and more effective enzymatic solutions to tackle the environmental issues caused by plastic pollution.

### Advancements in enzymatic stability through hydrogen bonding and electrostatic interactions

Significant progress has been achieved in enhancing the stability of protein structures with a dedicated emphasis on hydrogen bonding and hydrophobic interactions. The advancements in the strategic use of proline and its surrounding residues are very noteworthy. Prolines, known for their unique cyclic side chains, have played an important role in enhancing the structural rigidity and thermal stability of PET hydrolases.

The substitution of serine with proline in bacterial PET hydrolases such as Est119 from *Thermobifida alba* AHK119 and Cut190 from *Saccharomonospora viridis* AHK190 results in a notable enhancement in heat resistance and PET degradation efficiency. In the same way, the incorporation of proline into enzymes such as LCC^ICCG^ tetramer and *Thermobifida alba* cutinase has a notable effect on their melting temperature and boosts their hydrolytic activity toward PET. Enhancement of the hydrogen bonding network in enzymes has been a key area of study, and PETase is a prominent illustration of this. Modifications made to the flexible regions of these enzymes have led to the development of variants that exhibit enhanced rigidity and thermal stability.^176,180^ Notable examples include the IsPETase S121E/D186H double variant and the ThermoPETase triple variant. These variants exhibit higher melting temperatures and stronger binding to PET, which enhances their suitability for industrial applications.^181^ FAST-PETase mutants, such as S132E, D186H, R224Q, N233K, and R280A, have been found to exhibit exceptional kinetics and performance at elevated temperatures, surpassing the capabilities of the original enzyme in terms of hydrolysis efficiency. Crystal structure analysis of these mutants has confirmed their noteworthy contribution to enhancing the thermal stability of the enzyme.^182^

Nevertheless, challenges persist in breaking down highly crystalline plastics, which hinders the extensive use of these enzymes. Research has indicated that FAST-PETase can fully break down PET after thermal pretreatment, suggesting that these enzymes are successful in the recycling process.^182^ However, their use in the environmental degradation of crystalline polymers is still somewhat restricted.

### Enhancing enzymatic stability with glycosylation in microplastic degradation

Enhancement of the thermal stability of enzymes used in microplastic degradation has gained significant attention, and glycosylation has emerged as a promising technique in this regard. This process has demonstrated encouraging results in enhancing thermal stability, particularly when used with enzymes expressed in eukaryotic microbial cells. Understanding glycosylation, a crucial post-translational modification, is essential to maintain protein stability and prevent thermal aggregation.

An excellent illustration is the PET hydrolase LCC, renowned for its exceptional thermal stability. The expression of LCC in *Pichia pastoris* led to glycosylation, which resulted in an enzyme form that exhibits increased resistance to high-temperature aggregation. This variant of LCC has enhanced efficiency in breaking down PET at elevated temperatures.^183^ Nevertheless, achieving precise control over the glycosylation sites on the enzyme's surface poses a serious challenge, given its potential impact on the interaction of the enzyme with the PET substrate. Precise positioning of these glycosylation sites is needed to prevent negative impacts on the active site and overall functionality of the enzyme. Cutting-edge computational methods, such as GRAPE, have been employed to enhance enzymes such as IsPETase, leading to the development of variants like DuraPETase. These variants exhibit a remarkable increase in melting temperature, thereby enhancing their stability and effectiveness in degrading PET.^184^

In a previously reported study, neural networks were used to conduct extensive *in silico* mutagenesis and experimental validation. The aim was to develop enzymes with enhanced thermal stability. During this study, a number of mutations were discovered in PETase that greatly enhanced its performance in high temperature conditions. The enzyme FAST-PETase demonstrated a notable enhancement in the rate of hydrolysis when compared to its original form. While it is important to consider the potential impact on the catalytic efficiency of the enzyme,^182^ it is also beneficial to increase thermal stability. The function of the enzyme can be influenced by structural modifications in the active site. As an illustration, replacing Ala with Arg280 in PETase enhances the speed of PET degradation.

**Fig. 5:**
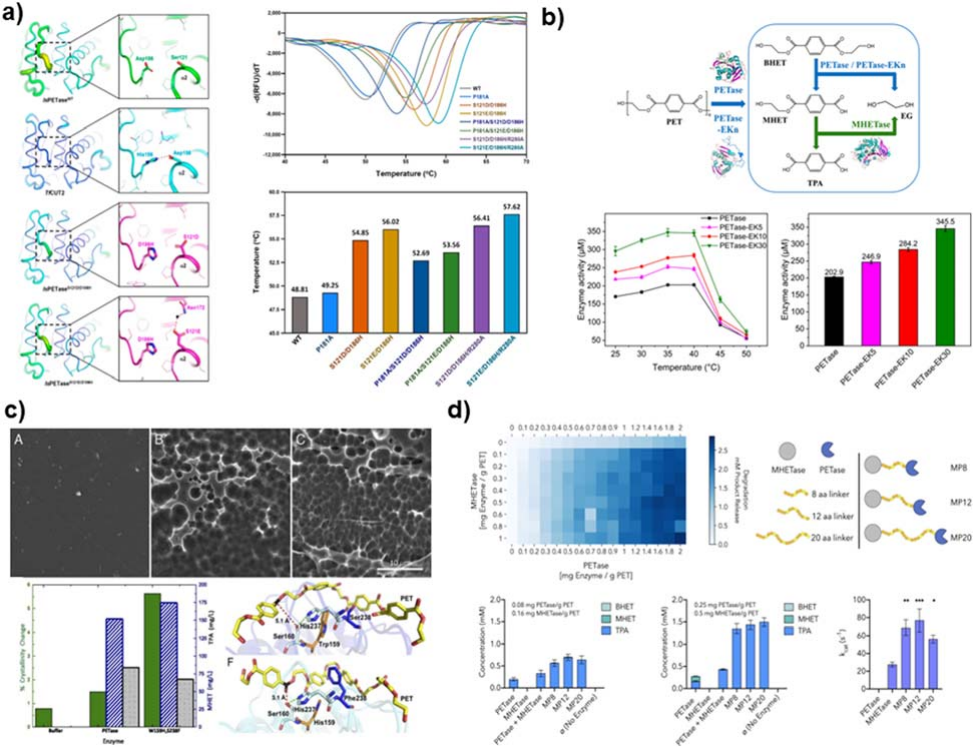
Strategies for enhancing thermal stability and catalytic activity of plastic degrading enzymes using protein engineering. (a) The IsPETase mutant, characterized by increased thermal stability and a higher *T*_m_ value compared to the wild-type IsPETase, achieves this improvement through the stabilization of the β6–β7 linked loop. Reproduced with permission from ref. 181. Copyright (2019) American Chemical Society. (b) Enhanced catalytic activity of a PETase-EKn variant with a more open substrate binding pocket resulting from C-terminal fusion of PETase with a zwitterionic polypeptide consisting of glutamic acid (E) and lysine (K) residues. Reproduced with permission from ref. 185. Copyright (2021) American Chemical Society. (c) PETase with a narrow active site due to the double mutation S238F/W159H shows a higher loss of crystallinity in PET and improved aromatic interaction with the substrate compared to wild-type PETase. Reproduced with permission from ref. 186. Copyright (2018) PNAS. (d) Synergistic depolymerization efficacy of MHETase-PETase, a chimeric enzyme linking the C terminus of MHETase to the N terminus of PETase, on PET films. Reproduced with permission from ref. 186. Copyright (2020) PNAS.

Glycosylation is a crucial element in enhancing enzyme thermal stability, and it is essential to carefully select a glycosylation site that does not disrupt the catalytic activity of the enzyme. A meticulous approach to enzyme modification is needed for the advancement of enzyme-based strategies for microplastic degradation (Fig. 5).

### Enhancing microplastic degradation through improved enzyme–substrate dynamics

The effectiveness of enzyme-based microplastic degradation relies heavily on the intricate interactions between enzymes and their specific substrates, particularly when it comes to PET hydrolases. This aspect is of the utmost importance in heterogeneous catalysis because it involves the solubility of the enzyme in an aqueous system, which is in stark contrast to the insolubility of the PET chains.^187,188^ This imbalance frequently results in substantial adsorption on the PET surface, which can have a negative impact on the catalytic efficiency of the enzyme.^189^ It is worth noting that the structure of PETase does not inherently facilitate substrate binding. This discovery has sparked a surge of scientific curiosity in hydrophobic polymers that resemble naturally occurring substances such as carbohydrate-active enzymes. The interaction between enzymes and PET substrates is mainly influenced by the electrostatic and hydrophobic interactions among the amino acid residues.^190,191^

The improvement of the interaction between enzymes and substrates is a main area of emphasis. One efficient approach is to alter the hydrophobic surface and/or electrostatic properties of the enzyme. Through an analysis of the charged and solvent-exposed amino acid residues of proteins, studies are striving to minimize the electrostatic repulsion between the PET substrate and the enzyme surface.^121,192,193^ These adjustments can enhance the binding affinity and boost the degradation efficiency of PET. These advancements are crucial in the development of enzyme-based methods for microplastic degradation and have a significant role in reducing the environmental impact of plastic pollution.

The enhancement of enzyme–substrate interactions is not just a scientific endeavor, but an important aspect of promoting environmental sustainability. Through careful optimization of these interactions, enzymes can be customized to better target and break down particular forms of microplastics, resulting in the higher overall efficiency of the degradation process. This approach shows immense potential in addressing the growing concern of microplastic pollution and offers a more efficient and eco-conscious solution to this worldwide problem. With the ongoing advancements in research, the potential for developing highly effective and specialized enzymes for microplastic degradation is growing.

### Enhancing the active site of PET hydrolase to enhance microplastic degradation

The enzymatic degradation of polyethylene terephthalate (PET) microplastics is intricately linked to the interaction between the active site of PET hydrolases and the PET substrate.^194^ In order to optimize the catalytic activity of these hydrolases, it is often necessary to make precise modifications in the active site region. These modifications may involve reconfiguring the substrate, adjusting the cofactor specificity, or introducing mutations in the active site that have been proven to greatly affect the overall reactivity.^195,196^ One important objective in PET-degrading enzyme engineering is to enhance the accessibility of the active site to the plastic surface.^197^ This is usually achieved by broadening the range of substrates that can bind to the enzyme. The approach used involved manipulating *Fusarium solani* cutinase to create the L182A mutant. This mutant was designed to have enhanced hydrolytic activity against PET by making specific amino acid modifications to expand the active site niche.^198^

Enzymes such as PETase, Cut190, MHETase, and *Pseudomonas aestusnigri* hydrolase have employed comparable techniques to selectively target residues in the substrate binding site by means of structural mutagenesis.^199^ Recent modifications have significantly enhanced the binding of PET substrate and minimized the interference caused by degradation byproducts. As a result, the efficiency of PET depolymerization has been greatly improved.^200^ Efforts are currently being made to enhance the hydrophobic characteristics of the binding site or optimize the active site. These efforts strive to enhance or diminish the bond of substances, ultimately resulting in more efficient PET degradation.^185,198,201–203^

In addition, modifying the structure of the active site of the enzyme can assist in reducing the inhibition caused by PET degradation intermediates or products.^204^ These different strategies work together to improve the interaction between PET hydrolase and its substrate for more efficient degradation of PET. This is a significant contribution to tackling the environmental issues associated with microplastics made from PET. The improvement of the active site of PET hydrolases is a crucial step in the development of more efficient enzymatic solutions for microplastic degradation (Table 2).

**Table 2:** Examples of different protein engineering strategies of plastic-degrading enzymes to improve thermostability and biocatalytic performance

Enzyme	Source	Substrate (ligand)	Engineering strategies	Mutation(s)/modification(s)	Biological effects (results)	Ref.
**Enhancing enzymatic thermal stability**						
Cut190	*Saccharomonospora viridis* AHK190	PET	Substitutions of amino acid through site-directed mutagenesis	S226P/R228S	Increase of *T*_m_ value by 3.7 °C with higher compared with the wild-type Cut190	122
IsPETaseTM	*Ideonella sakaiensis*	PET	Substitutions of amino acid through site-directed mutagenesis	S121E/D186H/R280A	Increase of *T*_m_ value by 8.81 °C at 40 °C and 14-fold improved PET degradation activity compared with the IsPETaseWT	181
Est1	*Thermobifida alba* AHK119	PET	Introducing proline and valine residues	A68V/T253P	The introduction of proline enhances heat resistance and activity in Est1 and Est119 compared to their respective enzyme types without proline	180
Est119						
LCC-G	Leaf and branch compost genome	PET	Introducing glycan	Glycosylation at N197 and N266	Resistance to thermal aggregation and improved PET hydrolysis activity at 10 °C higher than LCC enzymes	183
LCC	Leaf and branch compost genome	PET	Introducing disulfide bridge	D238C/S283C	Increase of *T*_m_ value by 9.8 °C higher than that of w/t LCC	179
Cut190*	*Saccharomonospora viridis* AHK190	PET	Introducing disulfide bridge	Q138A/D250C-E296C/Q123H/N202H	Improvement in PET film degradation by approximately 3 times and more than 30% at 70 °C, compared to the degradation of Cut190* at 63 °C	205
DuraPETase	*Ideonella sakaiensis*	PET	Accumulation of a substantial number of mutations using the GRAPE strategy	S214H-I168RW159H-S188Q-R280A-A180I-G165A-Q119Y-L117F-T140D	Maintains thermal stability at high temperatures of 60 °C for 3 days	184
ICCG variants	Leaf and branch compost genome	PET	Triple residues mutation	A59K/V63I/N248P	Higher *T*_m_ values (98, 98.9, 98.6 °C *vs.* 95.2 °C) of ICCG variants (RIP, KIP, KRP) compared with ICCG	176
				A59P/V63I/N248P		
				A59K/V63R/N248P		
HotPETase	*Ideonella sakaiensis*	PET	Induction of mutations by evolutionary pressure	21 mutations	Highest *T*_m_ value (82.5 °C) recorded to date among IsPETase derivatives	206
PES-H1^R204C/S250C^	Leaf and branch compost genome	PET	Introducing disulfide bridge	R204C/S250C	Increase of *T*_m_ value by 6.4 °C higher than that of w/t LCC	207
PETase^iS136E^	*Ideonella sakaiensis*	PET	Using the PROSS (Protein Repair One Stop Shop) approach, which recommends amino acid substitutions related to enzyme stabilization	S136E	Increase of *T*_m_ value by 1.1 °C and activity half-life *t*_1/2_ by 2.4 times higher than that of w/t IsPETase	181
Cut190*SS	*Saccharomonospora viridis* AHK190	PET	Multiple residues mutation	Q138A/D250C-E296C/Q123H/N202H/S226P/R228S	Increase of *T*_m_ value by 30 °C higher than that of Cut190* (Cut190^S226P/R228S^ mutant)	208
TfCut2^D204C/E253C^	*Thermobifida fusca* KW3	PET	Replacement of calcium binding site with disulfide bridge	D204C/E253C-	Increase of *T*_m_ value up to 94.7 °C (w/t TfCut2: 69.8 °C) and half-inactivation temperature up to 84.6 °C (w/t TfCut2: 67.3 °C)	209
IsPETaseTM^K95N/F201I^	*Ideonella sakaiensis*	PET	Amino acid substitutions through site-directed mutagenesis	K95N/F201I	Increase of *T*_m_ value by 5.0 °C higher than that of IsPETaseTM (IsPETase triple mutant)	210
TtC-G	*Thielavia terrestris* (TtC)	PET	Introducing glycosylation	Glycosylation at N127 and N195	Increase of *T*_m_ value by 3.0 °C higher than that of TtC-nonglycosylated forms	211
PET2 7M	*Ideonella sakaiensis* 201-F6	PET	Introducing disulfide bridge	R47C/G89C/F105R/E110K/S156P/G180A/T297P	Increase of *T*_m_ value by 6.7 °C higher than w/t PET2	212

**Improving interaction with substrate through modification of enzyme active site and surface**						
Cutinase variants	*Fusarium solani* pisi	PET	Expanding the active site using site-directed mutagenesis	L81A/N84A/L182A/V184A/L189A	Increase of hydrolytic activity by 4- and 5-fold on PET fibers compared with w/t cutinase	198
PE-H(Y250S)	*Pseudomonas aestusnigri* VGXO14^T^ genome	PET	Expansion of the active flanking niche site using site directed mutagenesis	Y250S	Increase of enzymatic activity by 3-fold on pNPB compared with PE-H	201
PETase^S238F/W159H^	*Ideonella sakaiensis* 201-F6	PET	Narrowing the substrate binding gap through site-directed mutation of active site residues	S238F/W159H	Increase of absolute crystallinity loss by 4.13% in PET films higher than that of w/t PETase	186
PETase^I179F^	*Ideonella sakaiensis*	PET	Improvement of enzyme hydrophobicity and steric hindrance through site-directed mutagenesis around the active site	R61A, L88F, I179F	Increase of *K*_cat_/*K*_M_ by 15.6-fold compared with w/t PETase	202
Tfu_0883^Q132A/T101A^	*Thermobifida fusca*	PET	Expansion of the active flanking niche site using site directed mutagenesis	Q132A/T101A	Increase of the amount of degradation product (TPA) by 10-fold and hydrolysis rate *k*_2_ by 10-fold compared with the w/t Tfu_0883	203
PETase-EK30	*Ideonella sakaiensis*	PET	Changing the substrate binding pocket of the PETase molecule to a more open structure using EKylation	EKylation	Increase of total released compounds by 11.5 times and enhance catalyst performance by 7.7 times compare with w/t PETase	185
Thc_Cut2	*Thermobifida cellulosilytica* DSM44535	PET	Increasing protein surface hydrophobic interactions through amino acid mutations located outside the active site	Arg29Asn or Ala30Val	Increase of *K*_cat_/*K*_M_ by 30-fold in PNPB substrate compared with w/t Thc_Cut2	192
PhaZ_RpiT1_	*Ralstonia pickettii* T1	PHB	Amino acid residue substitution to increase hydrophobic interactions between substrates	Y443F	Increase of maximum degradation rate up to 43 ± 3.7 mg min^−1^ compared with w/t 35 ± 0.5 mg min^−1^	213
del71Cbotu_EstA	Clostridium botulinum (Cbotu_EstA)	PET	Improving adsorption of the enzyme to the substrate by removing 71 residues at the N-terminus of the enzyme	Remove 71 residues from the N-terminus	Increase of total released compounds by more than 8 times, *K*_M_ by 4 times and *K*_cat_ by 2 times compared with the w/t enzyme	214

**Enzyme accessory module fusion and chimeric protein construction**						
PA_PBM	*Nocardia farcinica*	PU	Attaching the hydrophobic binding module of polyhydroxyalkanoate depolymerase (PBM) to polyamidase (PA)	Fuse PBM to PA C terminus	7.5 times more total released compounds and increase of enzymatic activity by 4-fold compared with w/t PA	215
Thc_Cut1_HFB	*Thermobifida cellulosilytica*	PET	Linking a partial region (P263 to P287) hydrophobin of cellobiohydrolase I from *T. reesei* to Thc_Cut1	Connecting hydrophobin to the N terminus of Thc_Cut1	Increase of hydrolysis product release rate by 16-fold compared with the w/t Thc_Cut1	72
Cutinase-CBM	*Thermobifida fusca*	PET	Fusing the carbohydrate binding module (CBM) of *T. fusca* cellulase Cel6A (CBM(Cel6A)) and *Cellulomonas fimi* cellulase CenA (CBM(CenA)) to the C terminus of *T. fusca* cutinase	Fusing the CBM module to the cutinase C terminus	Increase of the amount of released fatty acids up to 3-fold compared with the w/t Thc_Cutinase	216
Thc_Cut1-PBM	*Thermomyces cellullosylitica*	PET	Fusing the polyhydroxyalkanoate depolymerase of *Alcaligenes faecalis* to the C terminus of Thc_Cut1	Fusing the PBM to the C terminus of Thc_Cut1	Increase of the amount of released hydrolysis products by 3.75-fold compared with the w/t Thc_Cut1	217
IsPETaseEHA_CBM	*Ideonella sakaiensis*	PET	Fusing the Cellulose binding domain (CBM) to the IsPETaseS121E/D186H/R280A (IsPETaseEHA) variants C terminus of Thc_Cut1	Fusing the CBM to the C terminus of IsPETaseEHA	Increase of hydrolytic activity up to 71.5% higher than that of IsPETaseEHA	218
LCC^ICCG^-ChBD	Leaf and branch compost genome	PET	Fusing the chitin binding domain (ChBD) of *Chitinolyticbacter meiyuanensis* SYBC-H1 to the C terminus of the LCC^ICCG^	Fusing the ChBD to the C terminus of the LCCICCG variant	Increase of depolymerization performance by 11.6-fold compared with the LCC^ICCG^ without module	219
DSI-Tfuc2	*Thermobifida fusca*	PET	Fusing the Dermaseptin SI (DSI) to the N-terminus of *Thermobifida fusca* cutinase D204C/E253C (Tfuc2)	Fusing the DSI to the N terminus of the Tfuc2 variant	Increase of PET decomposition rate by 22.7 fold compared with the Tfuc2 variant	220
Lip–Cut	*Thermomyces lanuginosus*	PCL	End-to-end fusion of bifunctional lipase–cutinase (Lip–Cut)	Fusing the lipase and the cutinase	Increase of the weight loss of PCL film by 13.3 times, 11.8 times, and 5.7 times higher than that caused by Lip, Cut, and Lip/Cut blends, respectively	221
MHETase:PETase	*Ideonella sakaiensis*	PET	Covalently links the C terminus of MHETase to the N terminus of PETase	Fusing the MHETase and the PETase	Increase of hydrolysis product release rate by 3-fold compared with the single enzyme (MHETase)	222

### Enhancing enzyme surface properties to enhance efficiency in degrading microplastics

In the area of enzyme-based microplastic degradation, numerous endeavors have been undertaken to enhance the efficiency of plastic degradation. This involves modifying the surface properties and charge characteristics of the active site of PET hydrolases. The enhancement of the hydrophobicity of the active site has been shown to be a successful approach to improving binding to plastic substrates and consequently boosting degradation efficiency. As an illustration, when the cutinase Cbotu_EstA is adjusted,^214^ it reveals a greater portion of its hydrophobic surface. This leads to improved adsorption to PET substrates and an increase in hydrolytic activity. Nevertheless, an overabundance of hydrophobic residues may result in undesirable consequences like enzyme aggregation or structural instability.

In a previously reported study, certain mutations that greatly improve the activity of PETase were discovered. This was achieved with the use of molecular docking and crystallographic analysis. Several mutations, including R61A, L88F, and I179F, have been found to greatly enhance enzyme efficiency. In a similar vein, modification of the thermostable LCC and Tf Cut2 PET hydrolases by substituting His/Phe with Ser/Ile enhances their ability to break down PET at lower temperatures, resulting in more efficient depolymerization.^202^ The improvement of hydrophobicity can also enhance enzyme–substrate interactions. This was observed in the PHB-degrading enzyme from *R. pickettii* T1, where the substitution of serine and tyrosine with hydrophobic residues increased adhesion to the PHB surface. As a result, the efficiency of plastic hydrolysis was significantly improved.^223^

Recent developments in enzyme design have led to notable improvements in catalytic activity and a reduction in byproduct inhibition. For instance, the Tfu_0883 cutinase underwent a double mutation (Q132A/T101A), resulting in notable enhancements. The replacement of amino acid residues in the active site of the TfCut2 cutinase with residues from the LCC cutinase led to enhanced PET degradation at higher temperatures.^203^ Furthermore, the^192^ substitution of a mutation, Ile179, in the PETase enzyme with the more hydrophobic Phe resulted in an enhanced catalytic efficiency toward PET substrates at 30 °C.^202^ The modifications made enhanced the alignment of the binding sites and resulted in more robust interactions between the enzyme and substrate. These recent advancements underscore the significance of thoughtfully planned enzyme modifications in enhancing the breakdown of PET and other plastic materials. These strategic modifications in enzyme surface properties demonstrate promise for developing more efficient solutions to the urgent issue of microplastic pollution.^185,192,213^

### Improving enzyme functionality with the use of accessory binding domains

In order to enhance the efficiency of enzyme-based microplastic degradation, especially for PET, previous studies have investigated the integration of accessory binding domains that draw inspiration from the intricate structure of cellulases. These auxiliary modules, referred to as carbohydrate-binding modules (CBMs), are segments present in carbohydrate-active enzymes that facilitate the breakdown of natural biopolymers. The integration of CBMs into PET hydrolysing enzymes seeks to optimize the interaction of the enzyme with PET, leading to improved degradation efficiency. CBMs are highly regarded for their excellent compatibility with a wide range of natural polymers and synthetic plastics. Nevertheless, predicting the protein sequences that determine the function of PET-binding modules poses a serious challenge due to their inherent complexity.^224,225^

Notable advancements have been achieved in this field, such as the successful combination of a cutinase from *Thermobifida fusca* with CBMCenA from *Cellulomonas fimi* to enhance the breakdown of PET fibers.^216^ An alteration was made to CBMCenA by introducing a single tryptophan mutation. This modification aimed to enhance its adherence to PET fibers and enable more efficient enzymatic degradation.^226^ Moreover, the combination of Thc_Cut1 cutinase from *Thermobifida cellulosiliqua* and CBM trCBH from *Hypocrea jecorina* notably boosts the binding of the enzyme to the PET surface, thereby enhancing its capacity to break down the material. The combination of PET hydrolases and CBM has demonstrated encouraging outcomes in enhancing the efficiency of PET degradation in various PET feedstocks.^227^

Additional cutting-edge techniques being investigated involve the use of polyhydroxyalkanoate binding modules (PBMs), hydroponics, and amphiphilic anchor peptides for chimeric fusion. These methods focus on enhancing the attachment of enzymes to PET to improve the degradation of polyester–PU nanoparticles. As an illustration, the combination of hydrophobic, a protein with hydrophobic properties, and PETase has proven to be effective in improving the binding and degradation of PET films. These strategies, which aim to improve the binding ability of PET-degrading enzymes, show significant potential in optimizing PET binding and enhancing hydrolysis efficiency. This offers a fresh approach to tackling the issue of microplastic pollution.^72,215,217,219,220^

### Factors influencing the efficiency of enzymatic plastic degradation

In order to gain insights into the enzymatic degradation efficiency of microplastics, an extensive analysis was carried out to examine the distinctive properties of plastic polymers. The chemical structure, molecular weight, and crystallinity of these polymers play a crucial role in determining their degradation rate. Polymers containing ester bonds, such as polyester polyurethane, typically demonstrate greater biodegradability in comparison to polymers lacking these bonds.^228^ Biodegradation of high molecular weight plastics can be more challenging, but there are additives available that can assist in the process. The intricate composition of microplastics, with their symmetrical shapes, strong hydrogen bonds, and regular units, can often impede enzymatic degradation.^229^

Environmental factors are also influential in the degradation process. Temperature is important since elevated temperatures can speed up degradation and affect oxidation mechanisms. Accurate pH levels are also important in assessing the activity and growth of microorganisms that contribute to degradation. Additionally, different pH levels can affect the structure of plastic and its vulnerability to degradation. UV exposure and biodegradation are key factors contributing to the breakdown of plastic. Other factors, such as mechanical shredding, temperature, pH, and catalysts, also play a role in this process. Humidity plays a significant role in the biodegradation process. Higher levels of humidity generally support biodegradation, while excessive moisture can actually impede the process.^140,230–232^

The degradation kinetics of plastics are influenced by a variety of factors, resulting in a complex and multifaceted degradation process. This review highlights the significance of creating efficient enzyme-based strategies that are customized to the distinct characteristics of various microplastics to enhance their degradation efficiency.

## Tackling PET pollution with enzymatic solutions

### Degradation process of PET by hydrolytic enzymes

**Fig. 6:**
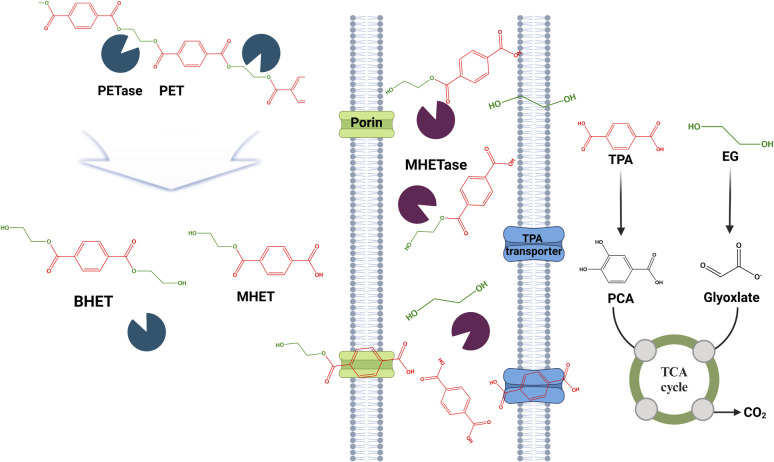
Proposed mechanism for PET degradation in *Ideonella sakaiensis*. The extracellular PETase enzyme catalyzes the hydrolysis of PET, yielding MHET as a byproduct. MHETase, predicted to be a lipoprotein, further hydrolyzes MHET into TPA and EG. Both TPA and EG can serve as energy sources for *Ideonella sakaiensis* and other microbes. Figures generated with BioRender (https://biorender.com/).

Polyethylene terephthalate (PET), a widely used synthetic plastic found in disposable beverage containers, has experienced a notable surge in global production. In 2013, a staggering 56 million tons of PET were manufactured.^233^ The durability of PET, stemming from its aromatic ring structure and ester bonds, plays a significant role in the issue of plastic pollution. Improper disposal of single-use plastics further worsens this problem. In contrast to other biodegradable polyesters such as polyhydroxyalkanoate, PCL, polybutylene succinate, and poly(butylene adipate-coterephthalate) (PBAT), PET is recognized for its resistance to natural degradation processes. Recent scientific advances have revealed fascinating insights into certain microorganisms capable of breaking down PET polymers.^34,234–236^ One such microorganism, *Ichneumonella sakaiensis* 201-F6, has demonstrated the ability to utilize the terephthalate component in its metabolic activity. This discovery offers a fresh perspective on the degradation of PET (Fig. 6).^34^

A study involving *Pseudomonas putida* GO16 highlights the significant progress made in biotechnology, specifically in converting PET into more environmentally friendly materials such as polyhydroxyalkanoates. This process, which uses pyrolysis, demonstrates potential for the recycling of PET waste.^237^ The degradation rate of PET films is influenced by various factors including crystallinity, purity, and the orientation of the polymer chains. These factors greatly affect the efficiency of the degradation process. PET microplastics present a serious environmental concern due to their potential effects on human health, specifically in relation to the endocrine system and estrogen regulation.^238,239^ This situation highlights the urgent demand for innovative and efficient degradation and recycling methods for PET and other synthetic polymers. Enzyme-based strategies for microplastic degradation have gained recognition as a viable solution, providing a sustainable approach to address the environmental consequences of PET pollution.

### Enhancing enzyme technology for efficient PET degradation

Remarkable advancements have been achieved in enhancing the performance of PETase, the key enzyme in the breakdown of polyethylene terephthalate (PET), in the area of enzyme-based microplastic degradation. Prior research has primarily concentrated on enhancing the interaction of PETase with PET substrates.^240,241^ As an illustration, PETase was modified with double mutations to enhance the efficiency of PET degradation. We conducted extensive research on double mutations in *Thermobifida fusca* to gain a deeper understanding of the enzyme's degradation capabilities.^203^

**Fig. 7:**
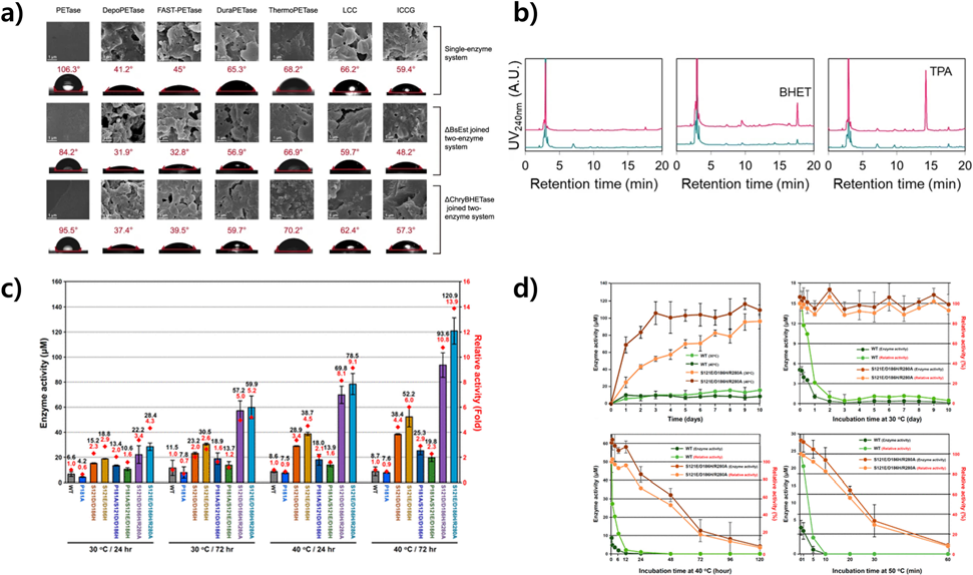
Increased PET decomposition efficiency by PETase deformation. (a) The SEM images (up panel) and water contact angle analysis (down panel) of the PET film in a single-enzyme degradation system, two-enzyme degradation system with ΔBsEst, and a two-enzyme degradation system with ΔChryBHETase after 48 h at 60 °C. Reproduced with permission from ref. 245. Copyright (2023) Springer Nature. (b) HPLC profiles of PETase powder incubation experiments: 2 weeks, 3 weeks and 4 weeks after incubation. Green and red lines indicate CC-124 wild type and CC-124_PETase #11 lysates. Reproduced with permission from ref. 246. Copyright (2020) Springer Nature. (c) PETase activity of the variants, PET degradation activity of IsPETaseWT and variants, the enzyme activity is the sum of MHET and TPA. Reproduced with permission from ref. 181. Copyright (2019) American Chemical Society. (d) Enzyme activity for 10 days and heat-inactivation experiment of IsPETaseWT and IsPETaseS121E/D186H/R280A. Reproduced with permission from ref. 181. Copyright (2019) American Chemical Society.

The activity of Cut190, a cutinase variant from *S. viridis*, was found to be influenced by the presence of Ca^2+^ in the binding site. Prior research has discovered three calcium binding sites in Cut190, each exerting distinct effects on the active site of the enzyme. By making modifications to these sites, the thermal stability of the enzyme was enhanced, and the degradation of PET was greatly increased.^242^ The main function of PETase is to transform PET into intermediate compounds such as mono-(2-hydroxyethyl) terephthalate (MHET) and bis-(2-hydroxyethyl) terephthalate (BHET). These compounds are subsequently broken down by MHETase into ethylene glycol (EG) and terephthalic acid (TPA). These products are subsequently involved in the tricarboxylic acid (TCA) cycle. PETase functions best in a pH range of 7–9 and maintains its stability in a pH range of 6–10.^243^ An optimal pH of 9.0 and a temperature of 30 °C were determined to be the most effective conditions for the variant of PETase. Efforts to enhance the stability of *Ideonella sakaiensis* PETase involved targeted genetic modifications to enhance its resistance to heat and prolong its effectiveness (Fig. 7).^244^

In a previous study, it was discovered that a specific mutation, R280A, demonstrated exceptional efficacy. This mutation showed enhanced activity and improved hydrolysis efficiency when BHET was used as a substrate. Additional examination of PETase resulted in the discovery of two mutations that enhance stability through the formation of hydrogen bonds: the substitution of serine at^247^ position 121 with glutamic or aspartic acid, and the substitution of aspartate at position 186 with histidine. The double mutation (serine 121 and aspartate 186) led to the development of a PETase R280A variant that exhibited a remarkable enhancement in degradation capacity, resulting in a 13.9-fold increase in efficiency.^182^ Recent research has also investigated the alteration of the protein structure to enhance plastic degradation. By integrating a poly-3-hydroxybutyrate (PBM) binding domain into the enzyme, its capacity to break down PET was greatly enhanced. With professional experimentation, it was discovered that incorporating a CBM domain into the mutant greatly enhanced the speed at which PET degradation occurred. In fact, the rate increased by 2.28 times when compared to the original variant. These advancements highlight the potential of engineered enzymes in effectively tackling the issue of PET microplastic pollution.^219,225^

## Approaches to address microplastic and nanoplastic pollution

### Exploring the environmental dynamics of micro- and nanoplastics

With the rise in plastic production worldwide, it is concerning to see how plastics are breaking down into microplastics due to various environmental factors such as ultraviolet light, temperature fluctuations, soil biological activity, and human involvement. Microplastics can undergo a process where they break down into even smaller particles called nanoplastics, which worsens the issue of plastic pollution. These microplastics can take the form of fibers, pellets, spheres, or flakes. The presence of micro- and nanoplastics is widespread in different environments, including landfills, wastewater systems, industrial and agricultural wastes, and polymer coatings.^248,249^ An in-depth understanding of the sources, properties, and distribution of these microplastic particles is crucial in the context of enzyme-based microplastic degradation strategies.^250^ This knowledge is vital for developing efficient measures to minimize their environmental impact. The extensive dispersion of these pollutants and their challenging degradation and recycling underscore the pressing need to tackle this worldwide environmental issue. An all-encompassing approach is required to take on the extensive dispersion of micro- and nanoplastics and minimize their effects on ecosystems.

### Biological effects of micro- and nanoplastics

Recent studies have uncovered the profound effects of microplastics and nanoplastics on the biological functions and health of a wide range of organisms. These studies have highlighted the negative impacts on ecosystem health and physiological processes of living organisms. They have emphasized the detrimental impact of large quantities of microplastics on the regular functioning of aquatic organisms,^251–253^ particularly fish. These minuscule particles, consumed through food and inhalation, result in harm to the tissues of fish. Giacomo Limonta's research on zebrafish revealed noteworthy alterations in the expression of immune response genes following exposure to low concentrations of microplastics. In Mehdi Banaei's research on *Cyprinus carpio*, significant findings were observed regarding gene expression and enzyme activity associated with oxidative stress and detoxification. These findings highlight the biochemical disruptions caused by microplastics in aquatic organisms.^254–256^

Microplastics present a significant danger to various forms of marine life, extending beyond just fish. Microplastics pose a serious ecological concern due to their capacity to adsorb heavy metals, harbor bacterial pathogens, carry multidrug-resistant *E. coli*, and act as a vector for persistent pollutants.^257–260^ The risks are further intensified by the combined effects of manufacturing chemicals and organic contaminants that are attached to microplastics. Microplastics also support the development of various microbial communities and create biofilms made up of algae, bacteria, and fungi. This occurrence has the potential to amplify the transmission of microbial pathogens and antimicrobial resistance.^261,262^

The potential health effects of microplastics on humans are currently receiving significant attention. It has been estimated that a considerable quantity of microplastics is consumed by humans annually, potentially resulting in various health issues including intestinal blockages, inflammatory reactions, and alterations in the gut microbiome. This growing research highlights the importance of further investigating the environmental and health effects of microplastics and nanoplastics.^263,264^ In particular, there is an urgent demand for the advancement and improvement of enzyme-based techniques to effectively break down these prevalent pollutants.

### Using microbial capabilities for plastic degradation

The search for microorganisms and enzymes capable of degrading plastics is gaining importance in the battle against plastic pollution. This requires the application of molecular cloning and culture techniques to improve the enzymatic and metabolic abilities of these microorganisms, thereby enhancing their potential for plastic degradation.^107^ The use of molecular biology tools, specifically polymerase-based rapid cloning, has been key in the discovery of polymer-degrading enzymes and the mapping of the genes responsible for these functions. These tools enable the manipulation of gene expression through genetic engineering to improve enzyme production, resulting in more efficient degradation in both the natural environment and composting facilities.^265^

Extensive microbial libraries have been developed to identify and validate microbes that excel at degrading plastics. Studies of the 16S rRNA gene in these libraries have yielded valuable insights into the microbial ecosystems linked to plastics. They have shed light on the interactions between these communities and factors such as substrate type, geographic location, and seasonal variations. The degradation of plastics by microbes is influenced by various factors, including the chemical composition of the polymer, environmental conditions, and the inherent characteristics of the microbes themselves. The chemical composition of the polymer is important in determining its biodegradability, with environmental conditions playing a supporting role in promoting degradation. Microbial enzymes are crucial in this process since they selectively target substrates for biodegradation. It is necessary to understand the metabolic pathways of microorganisms that efficiently degrade polymers to develop targeted strategies to combat microplastic pollution. This involves studying bacteria and fungi that play a key role in this process. This occurrence has the potential to amplify the transmission of microbial pathogens and antimicrobial resistance.^266,267^

The potential health effects of microplastics on humans are currently being closely examined. It is estimated that people consume substantial quantities of microplastics annually, resulting in potential health issues such as intestinal blockages, inflammatory reactions, and alterations in the gut microbiome. This emerging research highlights the importance of ongoing investigation into the environmental and health effects of microplastics and nanoplastics. There is an urgent demand for the advancement and improvement of enzyme-based techniques to effectively break down these prevalent pollutants.

## Conclusions

It is imperative that we address the pressing issue of plastic pollution caused by the widespread presence of microplastics and nanoplastics with immediate and efficient measures. This review has emphasized the significant role of enzymatic and microbial strategies in tackling these global environmental and health challenges. The use of enzymes such as PETase and MHETase, along with microbial degradation pathways, presents exciting possibilities for breaking down tough polymers such as PE, PET, and PS into more environmentally friendly substances. Despite the notable progress made in understanding and enhancing the capabilities of specific enzymes and microbes, there are still challenges that need to be addressed. Factors to consider are the efficiency of the degradation process, the scalability of these solutions, and the varying properties of plastic polymers. The significance of microplastics on the environment and health, specifically on marine life and human health, underscores the importance of implementing efficient degradation and recycling technologies. A combination of disciplines is needed to address plastic pollution and find effective solutions. Further studies and developments are necessary to enhance the effectiveness and real-world implementation of enzymatic and microbial degradation methods. Enzymatic and microbial strategies show great potential in addressing plastic pollution.

## Author contributions

J. C., H. K.; writing—review and editing, M. K., Y.-R. A.; conceptualization and methodology, S. Y., N. K. and S. Y. L.; visualization and investigation, J.-A. P. and S.-J. H; validation, K. S. L. and H.-O. K.; project administration. All authors have read and agreed to the published version of the manuscript.

## Conflicts of interest

There are no conflicts to declare.

## Supplementary Material
